# Detection of *Toxoplasma gondii* DNA in Malignant Breast Tissues in Breast Cancer Patients

**DOI:** 10.22088/acadpub.BUMS.6.3.190

**Published:** 2017-09-26

**Authors:** Narges Kalantari, Zeinab Ahangar Darabi, Sepideh Siadati, Novin Nikbakhsh, Masoumeh Ghasemi, Taraneh Ghaffari, Salman Ghaffari, Masomeh Bayani

**Affiliations:** 1 *Cellular and Molecular Biology Research Center, Health Research Institute, Babol University of Medical Sciences, Babol, Iran.*; 2 *Infectious Diseases and Tropical Medicine Research Center, Babol University of Medical Sciences, Babol, Iran.*; 3 *Department of Pathology, Faculty of Medicine, Babol University of Medical Sciences, Babol, Iran.*; 4 *Department of Surgery, Cancer Research Center, Babol University of Medical Sciences, Babol, Iran.*; 5 *Student Research Committee, Babol University of Medical Sciences, Babol, Iran.*; 6 *Department of Parasitology and Mycology, Faculty of Medicine, Babol University of Medical Sciences, Babol, Iran.*; # First two authors have equal contribution.

**Keywords:** Breast cancer, FFPE tissues, PCR, T. *gondii*, women

## Abstract

Breast cancer is the most prevalent malignancy in women throughout the world. Similar to other cancers, a strong relationship between breast cancer and environmental factors such as infectious agents has been reported. *Toxoplasma gondii *is a protozoan parasite which may play a role in cancer induction. The present study aimed to investigate a possible association between a history of *T. gondii* infection and breast cancer by detecting *T. gondii *DNA in malignant and non-malignant breast and lymph nodes tissues from breast cancer patients with latent toxoplasmosis. Formalin-fixed, paraffin-embedded (FFPE) tissue blocks from malignant/non-malignant breast and lymph nodes were obtained from twenty-nine breast cancer patients who were positive for anti-*Toxoplasma* antibodies (IgG). FFPE tissue blocks were deparaffinized using hot water method, and DNA was extracted. A conventional PCR analysis was performed to amplify partial regions of *T. gondii *B1 and REP-529 genes. Ninety-three samples from 29 patients were examined. All patients were negative for anti-*T. gondii* antibodies (IgM). *T. gondii* DNA was detected in 3 (10.3%) patients by PCR analysis of either B1 or REP-529 genes. These include two malignant breast and one normal lymph node samples. Sequence analysis of these genes showed a good similarity with previously published B1 and REP-529 sequences of *T. gondii* in NCBI GenBank. This study did not find any association between *T. gondii* infection and breast cancer. Furthermore, it is the first molecular identification of *T. gondii* in FFPE tissue samples obtained from breast cancer patients.

Breast cancer is the most prevalent malignancy in women both in the developed and the developing countries, with almost 1.7 million new cases diagnosed in 2012. Overall, breast cancer represents approximately 11.9% of all new cancer cases, 25.2 % of all cancers in women, and 6.4% of all death-related cancers. Although its mortality rate is less than the incidence, but it is considered as a general cause of cancer death in both developed and underdeveloped regions (1). Breast cancer is a complex and multifactorial disease, and there is a strong relationship between genetic and environmental factors (2).

There are several reports that consider the relationship between infectious agents and cancer. For example, the association of *Helicobacter pylori *to gastric extranodal marginal zone lymphoma (ENMZL) (3) or risks of cirrhosis and liver cancer in patients with *Hepatitis* virus infection have been reported by many researchers (4). Also, direct association between helminths infection including *Schistosoma haematobium*, *Opisthorchis viverrini *and *Clonorchis sinensis ,*and cancer has been reported (5). Furthermore, an association between *Cryptosporidium* and gastrointestinal adenocarcin-oma in humans and animal model has been observed (6, 7).

*Toxoplasma gondii* is an obligate intracellular coccidian parasite distributed widely throughout the world. The most common form of infection in humans is asymptomatic, but it causes opportunistic infections in immunocompromised individuals. Symptomatic infection is usually characterized by lymphadenopathy and reticular cell hyperplasia (8). *Toxoplasma* infection is also considered to have a role in cancer induction. For instance, a study reported that the risk of brain cancer in humans increases in patients with *T. gondii *infection, and another study showed that the mortality rates positively correlated with the seroprevalence of *T. gondii* (9, 10)*. *The latter study suggested that *T. gondii *should be further investigated as a possible oncogenic pathogen to humans (10). However, there are several studies indicating that the seroprevalence of toxoplasmosis is significantly higher in patients with cancer than non-cancer patients, including breast cancer (11-13). In contrast, our previous study demonstrated that seroprevalence rate of *Toxoplasma* infection in women with breast cancer was non- significantly higher than healthy individuals (14). The present study aimed to investigate possible association between *Toxoplasma* infection and breast cancer by detecting *T. gondii* DNA in malignant and benign breast tissues and lymph nodes in women with latent toxoplasmosis.

## Materials and methods

**Samples collection**

A total of 29 women with breast cancer positive for anti-*T gondii* antibodies (IgG) at surgical intervention were enrolled in the current study. Anti-*T. gondii* antibodies (IgG and IgM) were evaluated by a commercial ELISA kit (Genesis M, UK) based on the manufacturer’s instruction (14).

Formalin-fixed paraffin embedded (FFPE) tissue blocks from malignant and non-malignant breast tissues, and tumoral and non-tumoral lymph nodes were obtained from the pathology laboratory of hospitals affiliated to Babol University of Medical Sciences, Babol, Iran. Seven micrometer sections of each specimen were provided. Microscopic slides were prepared from all tissues, and then examined by a pathologist to distinguish malignant and normal tissues using histopatho-logical criteria.

**Deparaffinization of FFPE tissues**

The deparaffinization process was performed using 90 C sterile distilled water as described by Kalantari et al. (15). In brief, a small section of paraffin-embedded tissue was put in sterile microtube containing one ml hot sterile distilled water, and then the tube was left until the paraffin block meltsed completely, and the tissue became visible. The water was removed and the tissue was washed with hot water twice. The tissue was stored at-20 C until used.

**DNA extraction and PCR analysis**

Total DNA was extracted from the tissue using one- tube tissue DNA extraction kit (BIO BASIC INC, Canada) according to the manufacturer’ s instruction. *T. gondii* RH strain was used as a positive control. DNA was extracted from the tachyzoites by one-tube tissue DNA extraction kit (BIO BASIC INC, Canada) with modification of the kit instruction. Briefly, a suspension of tachyzoites containing 130 parasites per ml was centrifuged one minute at 12,000 g and the supernatant was removed. The pellet was mixed well with 50µl suspension of lysis buffer T and proteinase K. Proteinase K was diluted 1:20 in lysis buffer T. The quality and quantity of the extracted DNA was measured by a Nanodrop 2000C (Thermo Scientific, USA). The extracted DNA from patients’ tissues and the tachyzoites were stored at -20C until used. Water was used as negative control.

A conventional PCR analysis was performed to amplify a part of B1 gene and 529 repeat from *T. gondii. *The oligonucleotide primer pairs used in the current study were: B1, 5' GCAATCGATAGTTGA CCACG 3' (forward), and 5' CTGTAATGTGAT ACTGTGCCAT 3' (reverse), 529, 5' TGTGCT TGGAGCCACAGAAG 3' (forward), and 5' GCAG CCAAGCCGGAAACAT 3' (reverse) (16). The PCR condition was, initial hot start at 94 C for 5 min, followed by 40 cycles of 94 C for 30 s, 59 C for 30 s and 72 C for 30 s. The final extension was performed at 72 C for 10 min. PCR products were run in 2% agarose gel containing ethidium bromide stain and visualized by UV in a gel doc instrument. Furthermore, the quality of DNA was checked by human toll like receptor 4 as described in our previous work (15).

**DNA sequencing and phylogeny**

The sequencing was carried out by an automated sequencer (ABI 3730XL DNA, Bioneer, Korea) using 10 μl of the same forward and reverse primers that was used for the PCR analysis. The sequences were subjected to BLAST (http:// www.blast.ncbi.nlm.nih.gov). A multiple sequence alignment was also generated with a gap opening penalty of 10 and a gap extension penalty of 1 for the pair wise and multiple alignments, respectively.

Phylogenetic analysis was carried out by the Mega v.6. program using maximum likelihood method and bootstrap of 1000 replicates.

**Nucleotide sequence accession numbers**

The partial sequence of the B1 gene of *T. gondii* isolate has been deposited in the NCBI database under the accession no. KX866385.

## Results

Twenty-nine women with breast cancer were enrolled in this study. The mean age of the patients was 50.48± 11.97 years. All patients were positive for antibodies (IgG) against *T. gondii* and were IgM negative. The mean of IgG titer was 53.9±64.7 IU/ml ranging from 13 to 287 IU/ml.

**Fig. 1:**
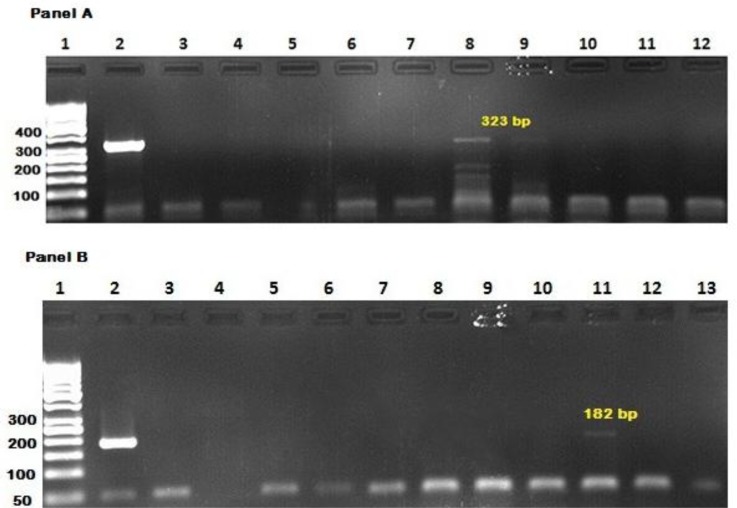
Gel electrophoresis of PCR product of *T. gondii* from malignant and normal breasts and lymph nodes. Panel A shows PCR analysis of B1 gene. Lane1: 50 bp ladder DNA size marker; lane 2: positive control; lanes 3-4: normal breast tissues; lane 5: negative control; lanes 6-8: normal lymph nodes; lanes 9-10: malignant breast tissues; lanes 11-12: normal breast tissues. Panel B shows PCR analysis of 529 gene. Lane 1: 50 bp ladder DNA size marker; lane 2: positive control; lane 3: malignant breast tissue; lane 4: negative control; lanes 5-7: normal lymph nodes; lanes 8-10: tumoral lymph nodes; lane 11: malignant breast tissue; lanes 12-13: normal breast tissues.

Ninety-three samples were examined in the current study. These include 27 (29%) normal breast tissues, 29 (31.2%) malignant breast tissues, 22 (23.7%) normal lymph nodes and 15 (16.1%) tumoral lymph nodes. Totally, DNA of *T. gondii* was detected either by PCR analysis of B1 gene or 529-gene in 3 (3.3%) tissue samples from three different cases (Figure 1). These include two malignant breasts identified by 529 and B1 genes, one sample by each pair primer, and one normal lymph node by B1 gene. Their anti-*T. gondii* antibodies (IgG) titer was 23.6, 16.5 and 30 IU/ml, respectively.

**Fig. 2:**
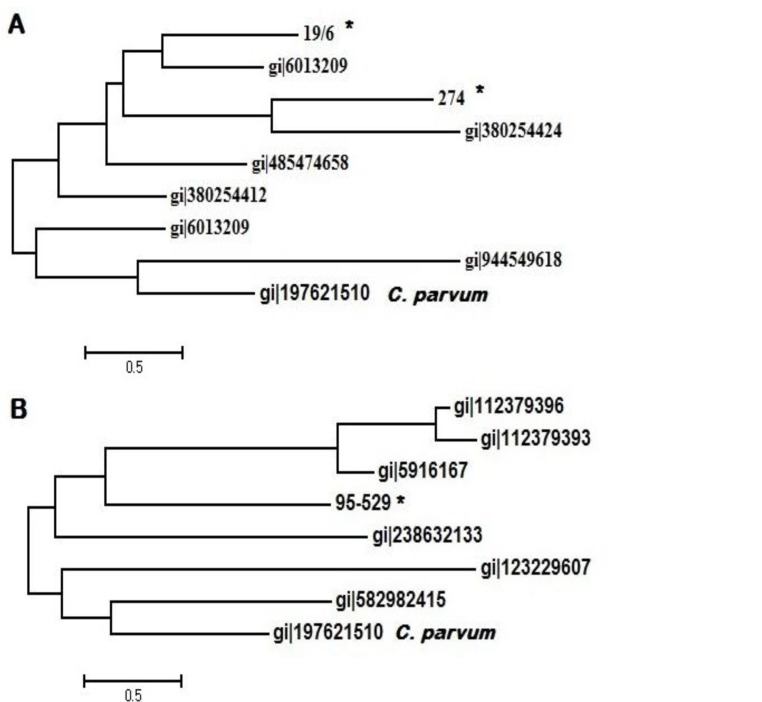
Phylogeny of *T. gondii* isolates by the Mega v.6. program using maximum likelihood method and 1000 bootstrap replicates based on B1 (panel A) and REP-529 (panel B) genes. * represent the positive samples from the current study.

Sequence analysis of the isolate obtained from malignant breast showed 88% identity, 75% coverage and E-value of 4e^-89^ with *T. gondii* B1 accession number AF1798710.1. The sample obtained from normal lymph node showed 96% identity, 77% coverage and E-value of 3e^-89^ with the accession numbers AF1798710.1 and AB703302.1. Multiple alignments revealed noticeable variation in the consensus sequences of the isolates obtained in the present study compared with previously published B1 gene. A phylogenetic tree of *T. gondii* B1 and some published sequences from NCBI GenBank is shown in Figure 2, panel A.*C. parvum* was used as out-group to anchor the tree.

Sequence analysis of the malignant breast tissue which was positive for repetitive fragment (529) showed 91% identity, 72% coverage and E-value of 1e^-34 ^with *T. gondii* repetitive DNA sequences (KF872166.1 and FJ656209.1). A phylogenetic tree of *T. gondii* B1 and some published sequences from NCBI GenBank is shown in Figure 2, panel B.*C. parvum *was used as out-group to anchor the tree.

## Discussion

Several studies have reported that the seroprevalence rate of *T. gondii* infection was higher in patients with different types of cancer including brain, hematologic and breast cancers (9-13). In addition, Shen et al., found *Toxoplasma* DNA in two cases with primary intraocular B-Cell lymphoma (17). Therefore, the possible role of *T. gondii* in inducing cancer is debatable. The current study was designated to investigate the probable association between a history of *Toxoplasma* infection and breast cancer by examining the presence of *Toxoplasma *DNA in malignant and non-malignant breast and lymph node tissues in seropositive patients.

The current study found no evidence showing an association between a history of *Toxoplasma* infection and breast cancer. However the DNA of the parasite was detected in three samples belonging to three different cases.

Detecting *Toxoplasma* DNA in solid human tissues, particularly in FFPE tissue blocks is very rare. For example, a study found *T.gondii* DNA in 42 out of 546 various tissue biopsies obtained from immunocompromised patients (18). There is no report on amplifying *Toxoplasma* DNA from human breast tissues with breast cancer. This may be due to low level sensitivity of PCR amplification of *Toxoplasma* DNA, regardless of the clinical condition consideration (19). It seems that successful amplification of *T. gondii* DNA even in blood samples in addition to the technique of PCR depends on the time of sampling post infection because of the transient nature of parasitaemia (20). However, in the current study, *Toxoplasma* DNA was amplified in three samples obtained from FFPE tissue blocks. We think that the method of deparaffinization plays an important role to gain positive amplification. Here, hot water was used instead of xylene to deparaffinize FFPE tissue blocks. This is a safer and easier method, and avoids multiple steps necessitating the use of multiple organic and non-organic solvents (15) which affect the quality of extracted DNA (21).

In the present study, PCR analysis of B1 gene and REP-529 was performed to find *Toxoplasma* DNA in breast cancer patients because they are multi-copy targets, and therefore are more sensitive in detecting *T. gondii* than those with single-copy targets (22). The findings obtained from the present study found that the B1 gene (two positive samples) was more appropriate than REP-529 (one positive sample) for detection of the parasite in FFPE tissue samples. This result is supported by the findings of Wahab et al., which demonstrated that the REP-529 was not amplified in all HIV patients and 4.8% false- negative results was observed (23). Althongh, there are several studies indicating that the REP-529 with a cryptic function, in comparison with B1 gene, is more sensitive and suitable for diagnosis of *T. gondii *(20, 22, 24).

Furthermore, PCR analysis of the positive samples either by B1 or REP-529, and three negative samples were repeated three times and the same results were obtained. PCR of REP-529 was negative for the samples that were positive for B1 and also B1 was not amplified in the positive sample by REP-529. The possible explanation is variation of whole or parts of B1 and REP-529 genes in different parasite strains. This explanation is supported by a study which demonstrated that the relative proportions of these genes differ among the isolates (23). Moreover, a range of factors may influence the results of the experiments such as the absence of target sequence, the choice of oligonucleotide sequences and possible polymor-phism (23).

Sequence similarity obtained by BLAST and phylogenic analysis of B1 gene showed that the isolate identified in lymph node may have different genotypes with the isolate in malignant breast tissue because they formed in different clades (Figure 2).

This study has limitations, such as to the small sample size for the detection of a significant difference between the compared tissues. and no information was available at the time of first *T. gondii* infection and cancer induction among the studied population.

In conclusion, the present study did not find any association between *T. gondii* infection and breast cancer. Furthermore, it is the first molecular identification of *T. gondii* in FFPE tissue samples obtained from breast cancer patients. Molecular studies using fresh frozen tissues may obviate the limitation in PCR caused by FFPE tissues and find a possible role for *T. gondii* infection in breast cancer or other malignancies. Besides, the development of tools or finding other suitable genomic sequences for sensitive and specific detection of *T. gondii* in patients with cancer is very important and helpful in obtaining precise results.
